# Therapeutic Effect of Botulinum Toxin A on Sensory Bladder Disorders—From Bench to Bedside

**DOI:** 10.3390/toxins12030166

**Published:** 2020-03-09

**Authors:** Yuan-Hong Jiang, Wan-Ru Yu, Hann-Chorng Kuo

**Affiliations:** 1 Department of Urology, Hualien Tzu Chi Hospital, Buddhist Tzu Chi Medical Foundation, Hualien 970, Taiwan; redeemer1019@yahoo.com.tw; 2 Department of Urology, Buddhist Tzu Chi University, Hualien 970, Taiwan; 3 Department of Nursing, Hualien Tzu Chi General Hospital, Tzu Chi Medical Foundation, Tzu Chi University, Hualien 970, Taiwan; wanzu666@gmail.com

**Keywords:** bladder, sensation, therapy, pathophysiology

## Abstract

Bladder oversensitivity arises from several different conditions involving the bladder, bladder outlet, systemic or central nervous system diseases. Increase of the bladder sensation results from activation of the sensory receptors in the urothelial cells or suburothelial tissues. Medical treatment targeting the overactive bladder (OAB) or interstitial cystitis (IC) might relieve oversensitive bladder symptoms (frequency, urgency and pain) in a portion of patients, but a certain percentage of patients still need active management. Botulinum toxin A (BoNT-A) has been demonstrated to have anti-inflammatory and antinociceptive effects in bladder sensory disorders and has been shown effective in the reduction of bladder oversensitivity and the increase of functional bladder capacity. For patients with OAB, urgency and urinary incontinence improved, while in patients with IC, bladder pain could be relieved in association with reduction of bladder oversensitivity after BoNT-A intravesical injection. Histological evidence has confirmed the therapeutic mechanism and clinical efficacy of intravesical BoNT-A injection on patients with OAB or IC. Bladder oversensitivity can also be relieved with the instillation of liposome encapsulated BoNT-A or low energy show waves (LESWs), which enable the BoNT-A molecule to penetrate into the urothelium and suburothelial space without affecting the detrusor contractility. Liposome encapsulated BoNT-A or combined LESWs and BoNT-A instillation might be future treatment alternatives for bladder oversensitivity in sensory bladder disorders.

## 1. Introduction

Bladder sensation arises from the urothelial cell and detrusor muscle stretching by signal transduction from peripheral receptors to the cerebral cortex. Many factors may contribute and effect the sensory transduction pathways, resulting in increased bladder sensation with or without associated symptoms such as urgency, urgency incontinence or bladder pain. The factors which will increase bladder sensation include aging, the urinary bladder, bladder outlet conditions, or systemic diseases. Currently, there is no definite definition nor specific medication targeting bladder oversensitivity. Bladder oversensitivity is usually existent with other lower urinary tract disorders (LUTD) such as bacterial cystitis, ketamine cystitis, interstitial cystitis (IC), bladder outlet obstruction (BOO), overactive bladder syndrome (OAB), idiopathic detrusor overactivity (DO), neurogenic DO (NDO); or systemic diseases such as diabetes mellitus, end-stage renal disease, or congestive heart failure [1]. Antimuscarinics or beta-3 adrenoceptor agonists have therapeutic effects on OAB and DO and also have some effect on reducing bladder oversensitivity. [2,3] Treatment of bladder oversensitivity should begin with treating the underlying causative diseases such as BOO, acute or chronic bladder inflammatory diseases, diabetes mellitus, or other systemic diseases. Previous studies of botulinum toxin A (BoNT-A) treatments for OAB, DO and IC have shown significant improvement of bladder oversensation and increased bladder capacity, in addition to the main target of urinary urgency or bladder pain. If medical treatment fails, intravesical BoNT-A might play a second- or third-line therapeutic alternative for bladder oversensitivity. This article reviews the pathophysiology, therapeutic mechanisms and treatment effects of BoNT-A on the improvement of the increased bladder sensation in bladder disorders, specifically focused on OAB, DO, IC and bladder oversensitivity. This review study searched relevant articles identified by a literature search using MEDLINE/PubMed. Key words included OAB, bladder oversensitivity, DO, IC, bladder sensation, botulinum toxin A, BOTOX, and BTX-A. References of retrieved articles were also hand searched to find additional articles related to the topic of this review. All trials examining the use of BoNT-A injections into the urinary bladder for the treatment of OAB, DO, IC and bladder oversensitivity, and the studies reporting the treatment outcome such as urodynamic parameters, urinary incontinence improvement, and adverse events were included in this review. 

## 2. Lower Urinary Tract Disorder and Bladder Oversensitivity

Lower urinary tract symptoms (LUTS) are highly prevalent and greatly impact the health-related quality of life and cause social economic burden, especially in elderly men and women [4,5,6]. The etiology of LUTS could be bladder outlet dysfunction or bladder dysfunction, including bladder oversensitivity and DO, or a combination of both bladder and bladder outlet dysfunctions. [7] Recent studies revealed the prevalence of OAB is high in Europe and the United States, as well as in Asian countries. [8,9] As most men with clinical OAB do not have urinary incontinence, they are frequently mis-diagnosed with bladder outlet obstruction (BOO) [10].

The bladder epithelium, known as urothelium, provides a passive barrier to prevent absorption of urine and its contents. Recent evidence suggests the urothelium might be a responsive organ with sensory and transducer functions. [11] The urothelial cells and suburothelial afferent nerves exhibit a number of common properties, including sensory receptors and ion channels. The local or systemic conditions which alter the afferent nerves or urothelial cells might contribute to the abnormal sensory of the urinary bladder. [11] Patients with several lower urinary tract disorders such as BOO, IC, ketamine cystitis, spinal cord injured (SCI), neurogenic bladder dysfunction, or systemic diseases such as diabetes mellitus, end-stage renal disease, or congestive heart failure may also have symptoms of OAB or bladder oversensitivity [12].

## 3. Pathophysiology of Bladder Oversensitivity

The pathophysiology of OAB and bladder oversensitivity is multifactorial. Occult neurogenic bladder, undetected BOO, provoked DO due to urethral incompetence, aging or diseases, chronic bladder ischemia, chronic bladder inflammation, central nervous system (CNS) sensitization, and autonomic dysfunction are possible etiologies of refractory OAB. [1] The bladder urothelium and afferent nerves express transient receptor potential vanilloid receptor 1 (TRPV1), the purinergic receptor P2X3, the sensory neuropeptides substance P, and calcitonin gene-related peptide (CGRP). [13,14,15] These receptors are believed to be involved in the afferent pathways that control bladder sensation and urinary volume reflexes [16].

Bladder sensation can be transmitted by the myelinated A-δnerves and unmyelinated C-fibers. In mammalian bladders after SCI, the unmyelinated afferent C-fibers are found to become predominant and mediate the voiding reflex. [17] Intravesical vanilloid treatment using capsaicin or resiniferatoxin had been found effective in SCI patients with NDO or idiopathic DO through acting on TRPV1 [18,19]. After intravesical instillation of resiniferatoxin, urinary frequency significantly decreased and maximal cystometric capacity increased, and patients with NDO became dry days after treatment. [20] In addition, many C-fibers in the bladder urothelium contain sensory neuropeptides which can modulate the micturition reflex and cause DO [21]. Since TRPV1 receptors are found on the afferent nerves and co-localized with P2X3 receptors and other sensory nerves expressing substance P and CGRP, desensitization of the TRPV1 by vanilloid treatment can also decrease bladder oversensitivity and DO [22,23,24].

The urothelium functions as a sensory organ which receives information from the urinary bladder content and responds to mechanical, thermal or chemical stimuli by releasing adenosine triphosphate (ATP), nitric oxide and acetylcholine (Ach). Changes in the external environment or direct insult on the urothelium produced by different bladder disorders may convey these signals to nerves, detrusor muscles, and transmit to the CNS, resulting in bladder oversensitivity or detrusor contractions. [25] The suburothelial sensory nerves of the urinary bladder are abundant with vesicles containing Ach and ATP, suggesting the bladder lamina propria also play an important role in the transmission of bladder filling and fullness in response to bladder stretch by activating P2X_3_ receptors [26,27,28]. When the urinary bladder is infected or under traumatic conditions, the production of ATP, CGRP and substance P acts on afferent nerves in an autocrine fashion to increase afferent nerve activity [29]. Recent studies also found a suburothelial nexus of myofibroblasts or interstitial cells which might be involved in the micturition reflexes [30]. These cells are closely linked by gap junctions and have a response to ATP in a mode similar to the activated ATP-gated P2Y receptors [31,32]. The Ach and ATP released from urothelial cells on bladder filling were noted in patients with aging, which also implies the main pathological mechanism of OAB and bladder oversensitivity in older people [33]. In addition, increase of stretch-activated ATP release was found in the cultured human urothelial cells from patients with IC and SCI, suggesting the involvement of these neurotransmitters in DO and bladder oversensitivity [34].

Bladder inflammation is commonly found in patients with OAB, IC, and systemic diseases and lower urinary tract disorders, resulting in bladder storage symptoms such as urgency and frequency [12]. In previous studies of IC, chronic inflammation leads to increase of urothelial cell apoptosis, lower adhesive protein E-cadherin and lower tight junction protein zonula occludens-1 expression [35]. The chronic inflammation in the IC bladders also inhibits the basal cell proliferation, causing defective apical cell maturation and impaired barrier function [36]. Further study of apoptotic markers such as Bad, Bax, and caspase 3 all increased in the IC bladder tissues, and the inflammatory signals such as p38 mitogen-activated protein kinase and tumor necrosis factor alpha were upregulated [37]. Chronic inflammation is also found in a large proportion of patients with OAB [38]. Chronic neural plasticity due to chronic inflammation and activation of sensory receptors may change the sensory afferent activity via influencing antinociceptive activity, resulting in bladder oversensitivity or DO. The urinary nerve growth factor (NGF) levels in patients with OAB, IC, or other types of bladder oversensitivity have been found to elevate, suggesting these different lower urinary tract disorders might share common pathways in increasing bladder sensation [39,40,41].

Based on the above evidence, the pathophysiology of bladder oversensitivity or IC syndrome might be sequentially developed by: (1) urothelial injury caused by bacterial cystitis, foreign body, instrumentation, or surgical trauma; (2) suburothelial inflammation developed after urothelial trauma, toxin or autoimmune response; (3) acute inflammatory cell infiltrations in the bladder wall after the acute injury; (4) chronic inflammatory reaction and scar formation in the bladder wall; (5) increased inflammatory reaction leading to neuroplasticity in the dorsal horn ganglia and corresponding sacral cord, resulting in lowering bladder sensation threshold and oversensitivity [42]. The insult of the bladder wall initiates an acute inflammatory process. The sensory impulse from the bladder wall also ascends to the corresponding cortical gyrus and produces frequency symptoms. Therefore, patients might have an early inflammatory reaction and characteristic IC symptoms, such as urgency, frequency and bladder pain. If the urothelial insult ceases, the urothelial cell regeneration will rebuild the defense mechanism and solves the inflammatory reaction to relieve bladder symptoms after treatment. However, if the urothelial insult continues, the inflammatory reaction might exacerbate not only at the bladder wall but also at the spinal cord or cortical gyrus, causing permanent inflammation printing [43]. Similar pathogenesis might also occur in OAB, in which bladder condition may progress from early stage (bladder oversensitivity) to late stage (urgency urinary incontinence) bladder conditions [44].

## 4. Therapeutic Mechanism of Botulinum Toxin A on Bladder Oversensitivity

BoNT-A has both motor and sensory effects in treating patients with DO or OAB. BoNT-A can inhibit the release of ACh and other neuropeptides from nerve terminals by cleaving the synaptosomal associated protein 25 kDa (SNAP-25), causing paralysis of the affected neuromuscular junctions [45]. In human bladders, Coelho et al. revealed that synaptic vesicle protein 2 (SV2) and SNAP-25 immunoreactive fibers were distributed throughout the suburothelium and muscle layer. Extensive co-localization of both proteins was noted in nerve fibers. SV2 is expressed more in parasympathetic fibers than in sympathetic or sensory fibers [46]. In nerve terminals, synaptic vesicles fuse with the presynaptic membrane where they release the neurotransmitter into the neuromuscular or neuroglandular junction. Intravesical BoNT-A injection relieves OAB symptoms as it enters bladder neurons binding to SV2, causing cleavage of SNAP-25 and preventing exocytosis of the neurotransmitter-containing vesicle at the nerve terminal [47,48]. Further studies revealed that after a single BoNT-A injection, cleaved SNAP-25 immunoreactive fibers were abundant throughout the guinea pig bladder tissue in the mucosa and muscular layer, significantly affecting the parasympathetic fibers. [49] However, because of the tight barrier function of the urothelium, intravesical instillation of BoNT-A cannot cleave SNAP-25 in nerve fibers. The bladder urothelium also expresses intracellular targets SNARE (Soluble *N*-ethylmaleimide-sensitive fusion Attachment protein REceptor) and binding receptor SV2 for BoNT-A uptake. BoNT-A has been shown to suppress the hypotonic-evoked ATP release from the cultured rat urothelial cells. BoNT-A injection can suppress sensory mechanisms and micturition reflexes after affecting the urothelial function of transmitter release [50].

Reduction of the expressions of TRPV1 and P2X3 on the suburothelial sensory afferents had been found in patients with DO treated with detrusor BoNT-A injections. [51] Patients also experience improvement of urinary frequency and reduction of the urgency severity after BoNT-A injection [52,53]. However, not all patients have similar sensory and motor therapeutic effects after BoNT-A injection. The reported success rates are between 60%-80% in patients with OAB [52,53,54,55,56,57,58,59]. Reduction of the urgency severity has been noted to be associated with long-term therapeutic efficacy after BoNT-A injections in patients with idiopathic DO [60]. Patients with both sensory and motor effects after BoNT-A injection showed a significantly better long-term therapeutic effect than the patients with motor or sensory effects alone. [60] Change of bladder capacity and increase of bladder fullness sensation after BoNT-A injections are also noted in these clinical trials. The improvement of bladder oversensitivity is likely to result from reduction of P2X3 and TRPV1 receptor expressions on the suburothelial afferent nerves [51].

In addition, BoNT-A injections for OAB and IC can effectively improve bladder sensory symptoms of frequency urgency and bladder pain in association with reduction of urinary NGF levels [61,62,63,64]. Recent studies also demonstrated that BoNT-A injection can inhibit cyclooxygenase-2 (COX-2) and prostaglandin E2 receptor 4 (EP4) expressions in the bladder tissue and block cyclophosphamide-induced bladder inflammation and overactivity. The increase of intercontractile intervals after BoNT-A injection indicates that BoNT-A can effectively improve both sensory and motor functions [65]. BoNT-A might also have a neuromodulatory or anti-inflammatory effect on the bladder wall in patients with OAB or IC, resulting in long-term sensory effect, possibly through CNS desensitization.

## 5. Clinical Effects of Botulinum Toxin A on Bladder Oversensitivity in OAB

The pathophysiology of OAB might be urotheliogenic or myogenic or could be due to neurogenic inflammation or BOO. As the pathophysiology of OAB might not clearly be determined before treatment, antimuscarinic agents or beta-3 adrenoceptor agonists usually cannot effectively treat all patients [66,67]. Recent investigation hypothesized that OAB might be a sensory disorder due to conditions activating the afferent nerve activities and resulting from the mild OAB subtype (hypersensitive bladder, frequency without urgency) to the moderate subtype (OAB dry, with urgency but no urgency incontinence) or severe subtype (OAB wet, with urgency urinary incontinence). [44] In a long-term, large-scale study, Wennberg et al. found the incidence of urgency incontinence and OAB increased over a 16-year span, with a certain percentage of patients having symptoms progress or regress [68].

Histological study revealed that chronic inflammation exists in half of the OAB bladders [38]. OAB had also been postulated as a subtype of neurogenic inflammation with vascular and non-vascular inflammatory responses through activation of bladder sensory afferents and release of sensory neuropeptides such as substance P and CGRP [69]. The inflammation might involve locally over-expressed suburothelial receptors such as TRPV1 and P2X3, or central inflammatory responses in the dorsal root ganglia [23,70]. Bladder inflammation leads to afferent nerve activation and results in long-term neuroplasticity which lowers the threshold of nociceptive and mechanoceptive afferent fibers, causing bladder oversensitivity. [71] The dynamic changes of OAB presentation with time might be due to different bladder conditions affecting afferent nerve activities [72].

Although many factors could cause OAB, the downstream pathophysiology of bladder oversensitivity and DO is similar. Through BoNT-A injection, the release of neuropeptides and neurotransmitters such ACh, ATP, substance P, or CGRP can be effectively reduced, causing impairment of sensory afferents and paralysis of the detrusor muscles. Treatment of OAB by intravesical BoNT-A injection has achieved satisfactory results in recent decades [52,58,73,74,75,76,77]. Currently, BoNT-A has been widely used in treatment of urinary urgency and incontinence in OAB patients’ refractory to oral medical treatment, and relief of frequency and bladder pain in patients with IC. The application of BoNT-A in OAB has been approved by the U.S. and European FDA and is listed as the third-line treatment in the AUA and EAU guidelines for OAB and urinary incontinence [78,79].

The dose of BoNT-A for OAB has been changed from 200 U or 300 U initially to the currently used 100 U [73]. A recent phase 3 clinical trial confirmed that the therapeutic results are similar between doses of 100 U and greater than 100 U [74,75]. BoNT-A at the dose of greater than 100 U increased the risk of adverse events of acute urinary retention and large post-void residual (PVR), as well as the incidence of urinary tract infection (UTI) [77,80]. Injecting BoNT-A at the trigone or bladder base for patients with OAB could effectively decrease the risk of acute urinary retention or large PVR without affecting the therapeutic effects of reduction of urgency and urgency incontinence [81]. The increased bladder sensation in OAB patients can effectively be decreased and bladder capacity increased after effective BoNT-A injection. Table 1 shows some representative study results on the improvement of bladder oversensitivity, reduction of frequency and urgency episodes, and increase of bladder capacity in OAB patients after different doses of BoNT-A injections.

## 6. Clinical Effects of Botulinum Toxin A on Bladder Oversensitivity in IC Patients

As BoNT-A injections can inhibit the release of ACh at the presynaptic neuromuscular junction and reduce the expressions of TRPV1, P2X3, CGRP and substance P in the urothelial cells and sensory fibers, this treatment has been enthusiastically applied in the treatment of IC [95]. Intravesical BoNTA injection can reduce bladder pain response and inhibit CGRP release from bladder afferent nerves in rat models [96]. The effect of BoNT-A on pain response to irritants might not only reduce bladder oversensitivity but also result in desensitization of the CNS in dorsal horn ganglia after long-term neuroplasticity [97].

An initial pilot trial was performed by Smith et al. who treated 13 IC patients with 100 U to 200 U of Dysport or BoNT-A into the trigone and bladder base and found subjective improvement in 69% of patients after treatment. They concluded that BoNT-A might have an antinociceptive effect on bladder afferent nerves [98]. Kuo et al. injected BoNT-A suburothelially to treat 10 women with IC and seven had symptom improvement. The cystometric bladder capacity significantly increased from 210 ± 63.8 mL to 287 ± 115 mL (*p* = 0.05), however, patients who responded to treatment also had dysuria after BoNT-A injection [95]. The therapeutic effect of BoNT-A on IC patients was further confirmed by Giannantoni et al., who treated 14 patients with 200 U of BoNT-A at 20 sites of the trigone and bladder base. After treatment, 12 patients (85.7%) had subjective improvement at one and three months, where urinary frequency and bladder pain decreased and functional capacity increased significantly [99]. However, the therapeutic efficacy decreased to none at the one-year follow-up [100]. Nevertheless, intravesical BoNT-A injection could reduce bladder pain, urinary frequency, and improve psychosocial functioning. Table 2 shows some representative study results of the changes of bladder capacity and improvement of bladder oversensitivity that are significant in IC bladders after different doses of BoNT-A injections. Due to significant therapeutic efficacy the application of BoNT-A on IC, it has also been recommended as the third-line for IC refractory to lifestyle modulation and medication for pain and urothelial glycosaminoglycan replenishment [101].

The dose of BoNT-A for IC has not been well determined. Pinto et al. used 100 U of BoNT-A to treat IC women by 10 trigonal injections and found subjective improvement in all patients at one- and three-month follow-up and the therapeutic efficacy remained for nine months in more than 50% of patients [104]. The therapeutic efficacy of 100 U of BoNT-A on IC patients was demonstrated in a large cohort. Significant improvement of interstitial cystitis symptom index (ICSI) and interstitial cystitis problem index (ICPI, 23.6 ± 5.9 versus 15.2 ± 8.5, *p* = 0.000), VAS (5.3 ± 2.2 versus 3.3 ± 2.4, *p* = 0.000), functional bladder capacity (136 ± 77.6 versus 180 ± 78.2, *p* = 0.000) and global response assessment (0.3 ± 0.8 versus 1.4 ± 1.0, *p* = 0.000) were shown at six months after 100 U of BoNT-A injection. BoNT-A injection has been proven to be a safe and effective procedure for relief of bladder pain and increase of bladder capacity in IC patients [105].

Kuo et al. conducted a randomized controlled trial (RCT) to compare the clinical effectiveness of 100 U or 200 U of BoNT-A intravesical injections followed by cystoscopic hydrodistention and hydrodistention alone in 67 IC patients. Among three groups, IC symptom score significantly decreased in all, but bladder pain VAS reduction and functional and cystometric bladder capacity increases were only noted in the BoNT-A groups at three months [102]. Recently, Pinto et al. compared the efficacy and safety of trigonal injections of onabotulinumtoxinA and saline in patients with IC. They found significant reduction of bladder pain in the BoNT-A group than the saline group at week 12. BoNT-A significantly improved IC symptom score and quality of life at all timepoints and the PVR did not increase at the endpoint, indicating that BoNT-A trigonal injection had safe and effective treatment outcomes without PVR increase [112].

In the histopathological investigations, Kuo et al. also investigated bladder tissue NGF mRNA at baseline and after BoNT-A treatment and found the NGF levels significantly increased in IC patients at baseline and decreased to normal in responders after BoNT-A treatment [61]. Shie et al. found that mast cell activity and apoptotic cell count did not decrease significantly; Bax and p-p38 but not tryptase content decreased significantly after a single BoNT-A injection [106]. After three repeated BoNT-A injections every six months, significant decrease of tryptase, Bax, p-p38 contents and apoptotic cell counts were noted, and SNAP-25 content in the bladder also decreased after BoNT-A injections. The immunohistochemistric improvements are also associated with clinical symptomatic improvements. This evidence further confirms that chronic inflammation and apoptotic signaling molecules in the IC bladder wall can be reduced significantly after repeated BoNT-A injections in IC bladders [42]. Repeat BoNT-A injections are needed to achieve a better and more durable success in the treatment of IC [107]. Repeated BoNT-A injections plus hydrodistention provides bladder pain relief and bladder capacity increase in responders. Through reduction of bladder suburothelial inflammation, the defective urothelial repair and improvement of cell differentiation ensue, leading to a healthy urothelium and thereby improving the clinical symptoms of IC [36].

## 7. Adverse Events after BoNT-A Injection for Sensory Bladder Disorders

Intravesical injection of BoNT-A has been demonstrated to be effective in treatment of NDO, IDO, OAB and IC, however, the high rates of treatment-related adverse events (AEs) still need attention. The most common AEs are large PVR, difficulty in urination, UTI and acute urinary retention [80]. For the male gender with IDO, a baseline PVR more than 100 mL with medical comorbidity and BoNT-A dose greater than 100 U are risk factors for AEs to increase after treatment [80]. The odds of increased PVR after BoNT-A injection were a nine-fold increase in comparison with placebo [92]. At the first treatment, the incidence of needing clean intermittent catheterization (CIC) was 35% and bacteriuria was 21% [113]. Poor efficiency, UTI and CIC-related issues are the most common causes of discontinuation of BoNT-A injections for DO over a seven-year span [114]. Large PVR after BoNT-A injection was significantly more in the frail elderly than elderly without frailty and younger patients; in frail elderly patients (60.7% v 39.7% v 35.7%, *p* = 0.018), the long-term success rate was significantly lower [82]. The incidence of AEs is closely related with the injected BoNT-A dose [77].

Regarding the AEs after BoNT-A injection for treatment of bladder and frequency in patients with IC, difficulty in urination and large PVR are the most common AEs [115]. The incidence of AEs in IC or OAB patients after BoNT-A injection is also related to the dose of BoNT-A. Injecting 100 U of onabotulinumtoxinA has less AEs than 200 U, while injecting 100 U of BoNT-A into the trigone showed no acute urinary retention and minimal AEs in both IC and OAB patients [102,104,112]. Interestingly, compared with IC, OAB patients suffered more frequently from AEs of hematuria, UTI, and large PVR (>200 mL) after BoNT-A injection, but less frequently from events of straining to void. OAB women had higher PVR volume and lower voiding efficiency than those in IC after BoNT-A injections, implying the bladder contractility of OAB patients are more susceptible to BoNT-A than IC patients [115]. However, in a randomized, double blind, placebo-controlled trial examining the effects of BoNT-A in patients with bladder oversensitivity, the significant increase in maximal cystometric capacity did not translate to clinical benefit in the majority of patients. Patients with bladder oversensitivity without DO had storage symptoms remain unchanged following BoNT-A injection and CIC was needed in three patients without clinical improvement [116].

## 8. Perspectives of Botulinum Toxin A on Sensory Bladder Disorders

Intravesical BoNT-A injection for OAB and IC have been demonstrated to be effective and well tolerated. However, large PVR and difficulty with urination remain problems to be solved [80]. Patients with OAB can benefit from BoNT-A for urgency and urgency incontinence relief, and patients with IC can have bladder pain relief after BoNT-A injection. However, for patients with bladder oversensitivity who do not have urgency or bladder pain, the adverse events that occurr after BoNT-A might not be acceptable [116].

Liposome instillation into the inflammatory bladder models have been shown effective to treat bladder hyperactivity in rat models [117]. As BoNT-A has a large molecular weight of 150 kDa, this neurotoxin cannot be delivered across the bladder urothelial cell membrane without an injection. Liposome encapsulated BoNT-A has also been demonstrated effective in improving the acetic acid-induced bladder hyperactivity in rat models [118]. The rats that received liposome encapsulated BoNT-A (Lipotoxin) showed significant decrease of response to acetic acid pretreatment without compromising the voiding function. Lipotoxin treatment also showed cleavage of SNAP-25, inhibition of CGRP from afferent nerves and blockage of hyperactivity induced by acetic acid. This evidence supports the idea that liposome can effectively carry BoNT-A across the urothelial barrier and has effects on bladder inflammation.

In a proof-of-concept clinical trial, Kuo et al. found that the BoNT-A binding protein SV2 could be demonstrated in the apical cells, urothelial cells and suburothelial tissues of the normal and OAB patients by immunohistochemistric or western blotting studies [119]. The changes of three-day urinary frequency (−6.50, IQR −18.3 to −0.25) and urgency (−12.0, IQR −20.3 to −2.75) significantly improved in the Lipotoxin group but not in the control group at one month post intravesical instillation of Lipotoxin containing 80 mg of liposome and 200 U of BoNT-A or normal saline [119]. A multicentric double-blind randomized trial using the same regimens also demonstrated that a single intravesical instillation of Lipotoxin could significantly decrease OAB symptoms and three-day frequency episodes (−4.64 for Lipotoxin and −0.19 for placebo, *p* = 0.0252) without adverse events of dysuria, large PVR or urinary tract infection [120].

Another recent study using Lipotoxin (containing 80 mg of liposome and 200 U of BoNT-A) for treatment of IC refractory to conventional medication revealed that a single Lipotoxin instillation was associated with a significant decrease in O’Leary-Sant symptom score (mean 7.38 ± 8.75), ICSI (4.00 ± 4.28), ICPI (3.35 ± 5.11) and VAS (1.64 ± 2.52); and an increase in the GRA score (1.35 ± 1.28). However, patients allocated to receive normal saline or BoNT-A in saline also had similar results [121]. Although the results of this study were negative, the effect of Lipotoxin on urinary frequency is similar to the results from Lipotoxin on OAB patients [120]. As patients with OAB or IC have symptom improvement, no difficulty with urination or large PVR after Lipotoxin intravesical instillation, this suggests that the penetration depth of BoNT-A carried by liposome is not as deep as that of a detrusor injection. The effect of BoNT-A in liposome might be limited to the urothelium. It seems rational to treat patients with bladder oversensitivity by liposome encapsulated BoNT-A rather than detrusor injection. However, the therapeutic duration of Lipotoxin instillation is short and the study was carried out in a small number of patients, indicating that the amount of BoNT-A protein diffused into the bladder urothelium was small. A large, randomized control trial is needed to clarify the effect of Lipotoxin on bladder oversensitivity.

Recently, low energy shock wave (LESW) has been enthusiastically applied in treatment of lower urinary tract disorders in animal models for bladder inflammation and overactivity [122,123]. LESWs suppressed cyclophosphamide-induced bladder pain, inflammation, and overactivity involved with the activation of IL6, NGF, and COX2 expression, indicating that it is a potential candidate for relieving bladder inflammatory conditions and overactivity [123]. LESWs also increased urothelial permeability, facilitated intravesical BoNT-A delivery and blocked acetic acid-induced hyperactive bladder [124]. In a preliminary clinical study including 15 patients with refractory OAB, Nageib et al. used intravesical instillation of 100 U of BoNT-A followed by LESWs (3000 shocks over 10 min) of exposure to the supra-pubic area. They found significant improvements in all OABSS domains and a total score at one and two months after treatment without an increase of PVR. [125] In the future, BoNT-A might be delivered via LESW to treat bladder oversensitivity without a need of intravesical injection.

## 9. Conclusions

The underlying pathophysiology of bladder oversensitivity in lower urinary tract disorders has not been well elucidated. However, chronic inflammation resulting in activation of the suburothelial sensory fibers, over-expression of the sensory receptors, and increased production of inflammatory proteins are likely to develop in patients with OAB, IC or bladder oversensitivity. Intravesical BoNT-A injection at the dose of 100 U can effectively reduce bladder inflammation, decrease the hyperactivity of sensory afferent nerves, restore normal urothelial barrier function, and desensitize the inflammatory printings in the central nervous system. The bladder oversensitive symptoms of OAB and IC can be improved after BoNT-A injections. However, large PVR and dysuria might develop after intravesical BoNT-A injection, especially in the elderly patients with low detrusor contractility. Liposome or LESWs can deliver BoNT-A across the urothelial barrier and have therapeutic effects on decreasing frequency and urgency episodes without compromising voiding function; these treatment modalities might have potential in treating patients with sensory bladder disorders.

## Figures and Tables

**Table 1:** The therapeutic effects of botulinum toxin A on the increase of bladder capacity in patients with overactive bladder syndrome.

Authors	Patients and OAB Subtype	Dose of BoNT-A	Change of Bladder Capacity after BoNT-A Treatment at 3 M	Reference
Kuo HC 2004	30 IDO + NDO	200(D)	223 ± 101 to 247 ± 96.3 mL	[54]
Schulte-Baukloh H 2005	44 OAB	200–300(D)	228 ± 19.2 to 305 ± 19.0 mL	[82]
Kuo HC 2005	20 IDO	200 (SU)	224 ± 125 to 315 ± 136 mL	[53]
Rajkumar GN 2005	15 IDO	300 (D)	MCC increased in 10 patients FDV increased in 12 patients	[58]
Smith CP 2005	110 IDO + NDO	100–300 (D)	153 ± 55 to 246 ± 64 mL	[83]
Popat R 2005	31 IDO	200 (D)	194 ± 24 to 327 ± 36.1 mL	[57]
Schmid DM 2006	100 OAB	100 (D)	246 to 381 mL	[84]
Kuo HC 2007	23 IDO + NDO	100 (SU)	185 ± 83 to 252 ± 159 mL	[85]
	25 IDO + NDO	150 (SU)	223 ± 133 to 303 ± 175 mL	
	27 IDO + NDO	200 (SU)	215 ± 124 to 315 ± 136 mL	
Sahai A 2007	16 IDO	200 (D)	MCC increased by 95.7 mL	[55]
Karsenty G 2007	12 OAB	200 (base)	MCC increased 162 to 370 mL	[86]
Kuo HC 2007	15 IDO	100 (SU)	243 ± 133 to 368 ± 132 mL	[87]
	15 IDO	100 (D)	260 ± 105 to 330 ± 116 mL	
	15 IDO	100 (base)	283 ± 167 to 318 ± 138 mL	
Rovner E 2011	57 OAB	50 (D)	MCC increased 50 ± 120 mL	[76]
	54 OAB	100 (D)	MCC increased 71 ± 129 mL	
	49 OAB	150 (D)	MCC increased 102 ± 127 mL	
	53 OAB	200 (D)	MCC increased 9 ± 1129 mL	
	53 OAB	300 (D)	MCC increased 131 ± 130 mL	
Tincello DG 2012	122 OAB women	200 (D)	Daily frequency 10.3 to 8.0	[88]
Fowler CJ 2012	57 OAB	50 (D)	UUI decreased 20.7/week	[89]
	54 OAB	100 (D)	UUI increased 18.4/week	
	49 OAB	150 (D)	UUI increased 23.0/week	
	53 OAB	200 (D)	UUI increased 19.6/week	
	56 OAB	300 (D)	UUI increased 19.4/week	
Denys P 2012	23 OAB	50 (D)	MCC increased by 38.4 ± 94.8 mL	[90]
	23 OAB	100 (D)	MCC increased by 85.5 ± 135.1 mL	
	30 OAB	150 (D)	MCC increased by 91.3 ± 125.2 mL	
Liao CH 2013	61 IDO frail	100 (SU)	247 ± 105 to 309 ± 133 mL	[91]
	63 IDO elderly	100 (SU)	266 ±124 to 309 ± 154 mL	
	42 IDO <65 yr	100 (SU)	254 ± 113 to 342 ± 103 mL	
Chapple C 2013	277 OAB	100 (D)	Frequency decreased by 19.7%	[75]
			Urgency decreased by 41.1%	
Nitti V 2013	267 OAB	100 (D)	Vol voided increased 41.1 mL (37.7%)	[74]
Mangera A 2014	IDO		MCC Improved by 58%	[92]
Wang CC 2016	21 DHIC	100 (SU)	Vol + PVR 255 to 365 mL	[93]
	21 OAB	100 (SU)	Vol + PVR 198 to 286 mL	
Onem K 2018	80 OAB	100 (D)	280 ± 134 to 330 ± 124 mL	[94]

OAB: overactive bladder, IDO: idiopathic detrusor overactivity, NDO: neurogenic detrusor overactivty, DHIC: detrusor overactivity and inadequate contractility, SU: suburethelial injection, D: detrusor injection, MCC: maximal cystometric capacity, FDV: first desire volume, MBC: maximal bladder capacity, Vol: voided volume, PVR: post-void residual.

**Table 2:** The therapeutic effects of botulinum toxin A on the increase of bladder capacity and decrease of bladder oversensitivity in patients with interstitial cystitis.

Authors	Patients and IC Subtype	Dose of BoNT-A	Change of Bladder Capacity after BoNT-A Treatment at 3 M	Reference
Smith CP 2004	13 IC	100–200	159 ± 39.9 to 250 ± 46.10 mL	[98]
Kuo HC 2005	10 non-ulcer	100 (SU)	210 ± 63.8 to 287 ± 115 mL	[96]
Giannantoni A 2006	14 IC	200 (D)	262 ± 34.8 to 342 ± 52.4 mL	[99]
Giannantoni A 2008	15 IC	200 (D)	256.4 ± 33.5 to 352.5 ± 50 mL	[100]
Kuo HC 2009	29 non-ulcer	100 (SU)	309 ± 135 to 388 ± 127 mL	[102]
	15 non-ulcer	200 (SU)	251 ± 86.7 to 407 ± 179 mL	
Giannantoni A 2010	13 IC	200 (D)	211.3 ± 48.9 to 341.4 ± 60.6 mL	[103]
Pinto R 2010	26 IC	100 (T)	106 ± 42 to 279 ± 82 mL	[104]
Chung SD 2012	67 IC	100 (SU)	261 ± 108 to 278 ± 144 mL	[105]
Kuo HC 2013	23 IC	100 x 3	277.2 ± 95.2 to 370.5 ± 173 mL	[106]
Kuo HC 2013	81 IC	100 x 3	270 ± 112 to 321 ± 160 mL	[107]
Lee CL 2013	30 non-ulcer	100 (SU)	305–316 to 379–395 mL	[108]
	10 ulcer IC	100 (SU)	142 to 110 mL	
Pinto R 2014	10 ulcer	100 (T)	Frequency 11.2 ± 2.4 to 7.9 ± 1	[109]
	14 non-ulcer	100 (T)	Frequency 10.3 ± 1.9 to 7.9 ± 0.9	
Wang J 2016	Non-ulcer	100–300	MBC increased by 50.5 mL	[110]
Kuo HC 2016	40 non-ulcer	100 (SU)	264.1 ± 120.1 to 332.0 ± 157.5 mL	[111]
Pinto R 2018	19 non-ulcer	100 (T)	Frequency 14.4 ± 6.3 to 9.5 ± 5.5	[112]

SU: suburothelial injection, D: detrusor injection, T: trigonal injection, MBC: maximal bladder capacity, IC: interstitial cystitis, BoNT-A botulinum toxin A.
